# The *FGG* c.952G>A variant causes congenital dysfibrinogenemia characterized by recurrent cerebral infarction: a case report

**DOI:** 10.3389/fneur.2024.1272802

**Published:** 2024-01-24

**Authors:** Anna Ying, Yuanlin Zhou, Chunyue Wang, Tao Wang, Xuan Zhang, Shanshan Wang, Shaofa Ke, Yuyan Bao, Yang Liu, Feng Wang

**Affiliations:** ^1^Department of Neurology, Taizhou Hospital of Zhejiang Province Affiliated to Wenzhou Medical University, Linhai, China; ^2^Key Laboratory of Digital Technology in Medical Diagnostics of Zhejiang Province, Dian Diagnostics Group Co., Ltd., Hangzhou, China; ^3^Department of Neurology, Saarland University, Homburg, Germany

**Keywords:** *FGG* gene, c.952G>A, congenital dysfibrinogenemia, cerebral infarction, fibrinogen

## Abstract

**Background:**

Congenital dysfibrinogenemia (CD) is a rare hereditary coagulation disorder resulting from mutations in fibrinogen genes. CD primarily presents with bleeding symptoms, but it can also lead to thrombotic events, including ischemic stroke.

**Case presentation:**

This report describes the case of a 52-year-old Chinese man who was admitted to the hospital twice due to recurrent cerebral infarction, characterized by sudden speech impairment and weakness in the right upper extremity. Brain MRI revealed multiple ischemic changes, predominantly in the left frontal and parietal lobes. Coagulation tests demonstrated reduced plasma fibrinogen (Clauss method), prolonged prothrombin time and thrombin time, and an elevated international normalized ratio. However, the ELISA assay indicated elevated levels of fibrinogen γ-chain protein. Despite a 2-month-old treatment regimen with aspirin, clopidogrel, and atorvastatin after the first hospitalization, the patient experienced a second ischemic stroke. Genetic analysis using whole-exome sequencing (WES) and Sanger sequencing identified a rare heterozygous missense variation, *FGG* c.952G>A (rs267606810), in both the stroke patient and his asymptomatic sister. Both individuals exhibited the same alterations in fibrinogen, characterized by reduced functional levels but increased antigenic protein. Subsequently, the patient was diagnosed with ischemic stroke associated with congenital dysfibrinogenemia.

**Conclusion:**

This case report expands the clinical phenotype spectrum associated with *FGG* c.952G>A (rs267606810) and underscores the significance of considering CD as a potential etiology for unexplained ischemic stroke, particularly in patients with a family history of coagulation disorders.

## Introduction

Congenital dysfibrinogenemia [CD; Online Mendelian Inheritance in Man^®^ (OMIM): #616004] is a rare hereditary coagulation disease, usually caused by mutation(s) in one of the genes *FGA*, *FGB*, or *FGG*, which jointly encode fibrinogen (Fib), a protein that plays a crucial role in coagulation (1, 2). Mutation(s) in the Fib gene may lead to changes in the structure or functional properties of Fib and further impair its ability to form stable blood clots. In CD patients, the synthesis of abnormal Fib generates a wide spectrum of clinical manifestations, ranging from asymptomatic forms to bleeding and/or thrombosis (1, 3). While bleeding tendency due to impaired clot formation is often associated with CD, a small proportion of CD patients may paradoxically have an increased risk of thrombotic events. Nevertheless, due to the variability of clinical presentation and the rarity of CD, clinicians are less likely to immediately consider CD as a cause of thrombotic events without further investigation. Several common coagulation function tests, such as Fib protein level and activity tests, active partial plasma prothrombin time (APTT), prothrombin time (PT), and thrombin time (TT), are the preferred methods for the diagnosis of coagulation disorders. However, these tests are of limited value in the etiological diagnosis of CD. In addition, interpretation of laboratory results for coagulation disorders can be complex, and misinterpretation of results often causes misdiagnosis or delayed diagnosis of CD, ultimately resulting in unnecessary or inappropriate treatments and an increased risk of fatal complications such as excessive bleeding, recurrent thrombotic events, and multiple organ failure (3–6). As CD is a genetic disease, genetic testing is often necessary for the definitive diagnosis and appropriate management of CD, especially when there is a suspected underlying genetic predisposition to unexplained thrombosis.

We report here the case of a 52-year-old male CD patient with abnormal coagulation function and recurrent cerebral infarcts. Through whole-exome sequencing (WES) analysis, we identified a heterozygous missense variant c.952G>A in the *FGG* gene as a potential genetic causative factor.

## Case presentation

### Case overview

The patient was a 52-year-old Chinese man who was admitted to the hospital due to two cerebral infarctions. Initially, a sudden speech disorder was the main cause of hospitalization. Physical examination at that time revealed only Broca’s aphasia-like symptoms. Coagulation tests indicated a coagulation disorder, which was characterized by a decrease in the plasma level of Fib as determined by the Clauss method, an increase in Fib plasma concentration as detected by ELISA, and prolongation of PT and TT. In addition, brain imaging examinations revealed ischemic changes in multiple brain regions. As a result, the patient was diagnosed with unexplained multiple cerebral infarction and was treated with aspirin, clopidogrel, and atorvastatin. Although the short-term treatment alleviated his speech disorder, it failed to prevent the recurrence of the cerebral infarction. A second ischemic stroke manifested itself in the form of mild aphasia and weakness of the right upper extremity. Through family screening and genetic testing, we identified a rare heterozygous missense variant *FGG* c.952G>A (rs267606810) in the patient and his asymptomatic sister, both of whom exhibited the same alterations in Fib, characterized by reduced functional levels (Clauss method) but increased antigenic protein (ELISA assay). Taking the findings together, the patient was definitively diagnosed with CD. Finally, the patient underwent long-term therapy with clopidogrel and atorvastatin, which has so far brought about a satisfactory recovery from cerebral infarction and the associated neurological symptoms.

### Initial hospitalization

**Figure 1:**
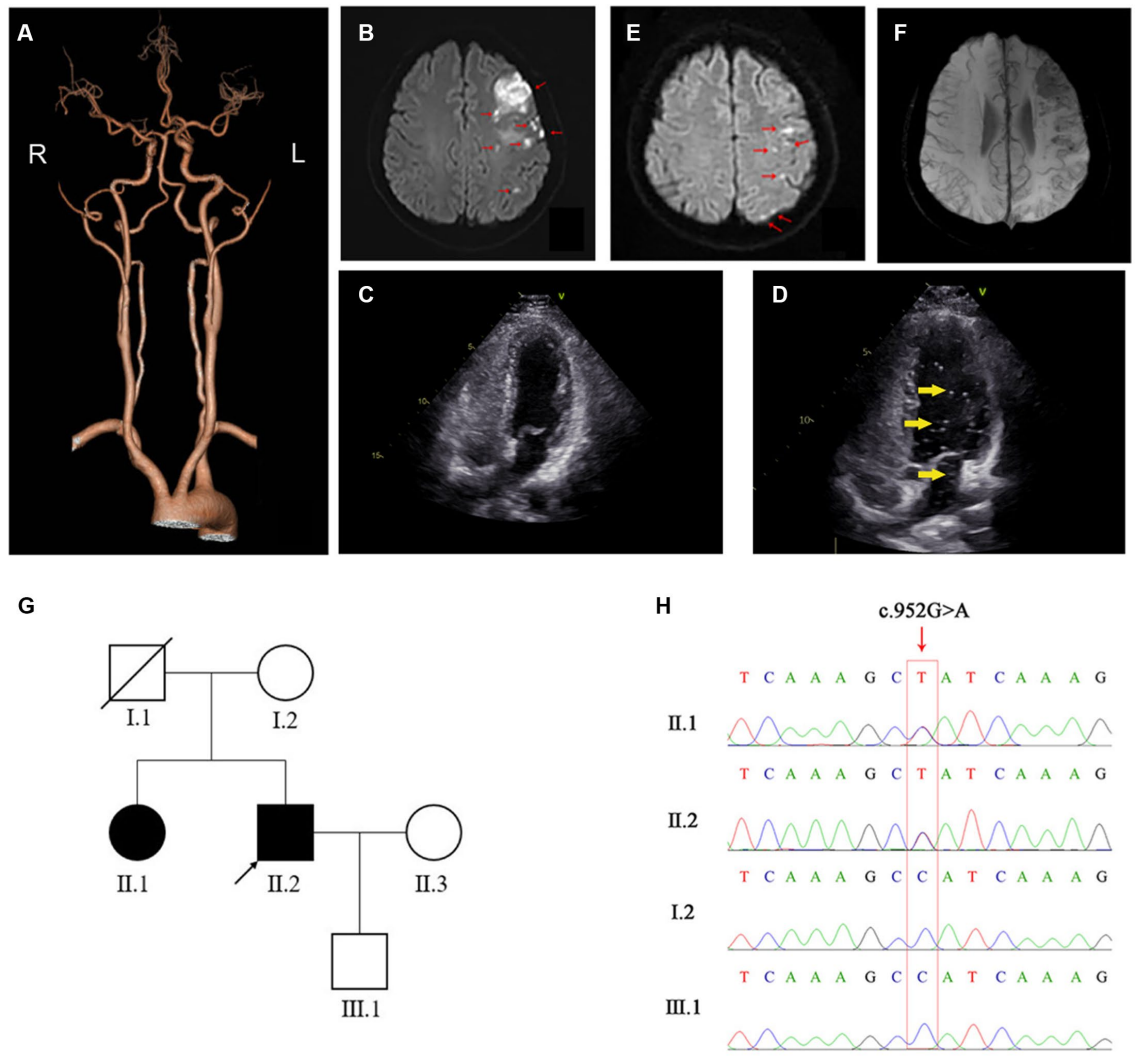
Imaging and genetic results for the patient. **(A)** Representative CTA image of the patient’s brain. **(B)** On first admission, DW-MRI of the brain shows high signal in the left frontal lobe, the parietal lobe, the occipital lobe, the basal ganglia, and corona radiate, as indicated with red arrows. **(C,D)** Agitated-saline-contrast echocardiography of the reported patient **(C)** and a positive control patient with patent foramen ovale (PFO) **(D)** show bubbles (indicated by yellow arrows) in the left ventricle of the PFO patient but not in the stroke patient (our case). **(E)** On second admission, DW-MRI of the brain shows high signal in the left frontal and parietal lobes, as indicated by red arrows. **(F)** SWI-MRI of the brain shows low signal in the left frontal lobes, the parietal lobes, and the left basal ganglia, where the lesions were located on first admission. **(G)** Pedigree analysis of the patient’s family. The patient and his older sister, who both carried *FGG* c.952G>A, are shown in solid black. Healthy family members are shown in hollow white. The patient, i.e., the proband, is marked with an arrow and his deceased father with a diagonal slash. **(H)** Sanger sequencing confirms the presence of *FGG* c.952G>A in the patient and his older sister, and the absence of *FGG* c.952G>A in his mother and son.

A 52-year-old man was admitted to our hospital on 30 April 2022 due to a sudden speech disorder that had persisted for 17 h. On admission, his blood pressure was 118/69 mmHg, and his heart rate was 58 beats per min. He was conscious and had normal facial expressions. The bilateral pupils were equal in size, round (diameter approximately 3.0 mm), and reacted to light. Notably, he exhibited Broca’s aphasia-like symptoms with repetition of words or simple phrases, but not fluent grammatical sentences, and he was aware of his own speech impairment. Nevertheless, the ability to understand was not impaired. Physical examinations showed normal systemic motor and sensory functions and normal muscle strength (grade V) in four limbs, and neither pyramidal signs nor meningeal irritation signs were observed. The National Institutes of Health Stroke Scale (NIHSS) score was 4, which indicated minor to moderate stroke. Brain computed tomography angiography (CTA) showed patchy hypointense signals in the left frontal lobe. The distal branches of the left anterior cerebral artery were sparser than those of the contralateral artery (Figure 1A). Diffusion-weighted magnetic resonance imaging (DW-MRI) further revealed ischemic changes in multiple brain regions, including the left frontal lobe, the parietal lobe, the occipital lobe, the basal ganglia, and the corona radiata (Figure 1B). Vascular ultrasound showed intima–media thickening of the right carotid artery and bilateral lower limb arteries. A complete blood count showed a high platelet count (366 × 10^9^/L), leucocyte count (12.5 × 10^9^/L), and absolute neutrophil count (9.0 × 10^9^/L), and a low erythrocyte count (4.23 × 10^12^/L). Importantly, laboratory tests showed reduced activity of plasma Fib (0.6 g/L, Clauss method), prolonged PT (16.1 s) and TT (26.6 s), and an elevated international normalized ratio (INR; 1.34), although APTT (32.7 s) and D-dimer (0.19 mg/L) were within normal reference ranges (Table 1). Moreover, plasma anticardiolipin antibody, antithrombin antibody, homocysteine, glycated hemoglobin, triglyceride, and cholesterol were normal. Electrocardiogram and cardiac ultrasound revealed normal cardiac function. Echocardiography with agitated saline contrast, also known as a bubble study, showed no bubbles within the left heart (Figure 1C). As a positive control, bubbles were visualized in the left heart within 3–5 beats in a patient with a patent foramen ovale (PFO) (Figure 1D). Abdominal ultrasound also showed no obvious abnormalities. Kidney and liver function were normal.

**Table 1:** Results of coagulation function tests in the patient and his older sister.

Member	Date	PT (s)	INR	APTT (s)	TT (s)	D-dimers (mg/L)	Fibrinogen (g/L)	
							Clauss	PT
Normal range	/	11.0–14.5	0.8–1.2	28.0–42.0	14.0–21.0	0–0.5	2.0–4.0	2.0–4.0
II.2	2022.04.30	16.1	1.34	32.7	26.0	0.19	0.60	/
	2022.06.17	16.1	1.33	34.2	25.3	0.10	0.45	3.02
II.1	2022.10.24	15.4	1.24	34.7	28.1	/	0.60	/

The patient was diagnosed with unexplained multiple cerebral infarction. As there was no indication for interventional therapy, he was treated with dual antiplatelet and hypolipidemic therapy for 10 days, including aspirin (100 mg, q.d.), clopidogrel (75 mg, q.d.), and atorvastatin (20 mg, q.d.). He was subsequently discharged with significant relief from Broca’s aphasia-like symptoms. After discharge, clopidogrel was continued for a further 11 days, and aspirin and atorvastatin were taken regularly.

### Readmission to hospital

On 16 June 2022, the patient was readmitted to the hospital due to mild aphasia and persistent weakness of the right upper limb for 29 h. On the second recording, his consciousness, pupils, blood pressure, and heart rate were normal, but he showed mild symptoms of aphasia. Strikingly, his right nasolabial sulcus was flat, and his mouth was tilted to the left. The muscle strength of his right arm was grade IV, and the other limbs were grade V. DW-MRI of the brain showed new ischemic lesions located mainly in the left frontal and parietal lobes, while encephalomalacia of previous ischemic areas was observed (Figure 1E). Susceptibility-weighted imaging (SWI) further revealed cerebral microbleeds and remote hemorrhage in the left frontal and parietal lobes and in the left basal ganglia (Figure 1F). Again, routine blood tests showed a high platelet count (385 × 10^9^/L) and low erythrocyte count (4.19 × 10^12^/L). Importantly, the PT-derived method detected a normal concentration of plasma Fib (3.02 g/L; Table 1), which was dramatically higher than the concentration detected by the Clauss method. The PT-derived/Clauss ratio of plasma Fib was 6.71. In addition to the previously observed abnormalities of common coagulation markers, we found an increased plasma level of von Willebrand factor (vWF) antigen (182%; normal range: 77.9%–137.1%), while plasma levels of protein S, protein C, antithrombin III, lupus anticoagulant, and fibrin/Fib degradation products (FDPs) were normal. Moreover, the activity of coagulation factor VIII was increased, while plasminogen (PLG) activity was normal. As the antiplatelet agents had been unable to prevent the recurrence of cerebral infarction, the patient was treated with atorvastatin and the anticoagulant rivaroxaban (15 mg, q.d.). However, the 2 days course of treatment with rivaroxaban led to nosebleeds, so rivaroxaban was discontinued. The patient then took clopidogrel and atorvastatin for a further 10 days, which gradually improved his aphasia, abnormal facial expression, and right arm weakness. After discharge, clopidogrel and atorvastatin were taken regularly, and strict follow-up was required. No further neurological symptoms had occurred at the time of writing of this article.

### Cascade genetic testing

The patient stated that he had no family history of neurological, hemorrhagic, and/or thrombotic diseases. Nevertheless, his asymptomatic older sister (55 years old) underwent the usual coagulation function tests, which also revealed a reduced plasma level of Fib (Clauss method), prolonged PT and TT, and an increased INR (Table 1). Other family members refused coagulation function tests. To determine the genetic etiology, we collected peripheral blood genomic DNA from the patient and performed WES analysis using standard protocols. After filtering common variants and variants that were irrelevant to neurological and coagulation disorders, we identified a rare heterozygous missense variant in exon 8 of the *FGG* gene, i.e., *FGG* c.952G>A (rs267606810). By Sanger sequencing, we further confirmed the presence of this *FGG* variant in the patient and his sister, but not in his healthy mother or son (Figures 1G,H).

*FGG* c.952G>A (rs267606810) leads to the replacement of non-polar and aliphatic glycine with polar and uncharged serine at position 318 in the C-terminal of the Fib γ-chain, which plays a crucial role in binding to the N-terminal of the Fib α-chain. *FGG* c.952G>A was predicted by REVEAL, ClinPred, SIFT, and PolyPhen2 to be highly detrimental to the function of the encoded protein. This missense variant has been reported in a patient associated with thrombotic disease (7). In the ClinVar database, *FGG* c.952G>A is defined as a variant of undetermined significance.^1^

Notably, ELISA assay using a human Fib γ-chain ELISA kit (Cat No.: JL19902, Jianglai Biological, China) further revealed that the patient and his sister had similar plasma levels of Fib γ-chain, which were higher than those of the patient’s healthy wife and five healthy control subjects (Table 2), suggesting that *FGG* c.952G>A may cause a compensatory increase in abnormal Fib γ-chain. Considering these genetic results, we speculate that *FGG* c.952G>A may contribute to the pathogenesis of the patient’s coagulopathy and recurrent cerebral infarction. Finally, a definitive diagnosis of CD was made for this patient.

**Table 2:** Plasma levels of Fib γ-chain.

Member	Fibrinogen γ-chain (pg/mL)
II.2	3.37
II.1	2.72
II.3	0.91
Control (*n* = 5)	2.02 ± 0.43

## Discussion

CD is a clinically heterogeneous hereditary disease that is primarily associated with bleeding tendency rather than thrombotic events. In a large cohort study with 102 Chinese CD patients, thrombotic events occurred in only 4/102 (3.9%), and the remaining patients either were either asymptomatic (68.6%) or showed bleeding tendency (27.5%) (8). CD-related thrombosis occurs in all blood vessels, but cerebral vessels are less frequently affected than vessels in the legs or lungs (8, 9). Cerebral thrombosis can lead to cerebral infarction, resulting in symptoms that are not specific to CD, such as severe headaches, seizures, visual and speech disorders, and other neurological deficits. Because of these overlapping symptoms and its rarity, a cerebral infarction caused by CD is difficult to distinguish from one with other causes.

Clinical manifestations of CD largely depend on the specific location(s) of Fib gene variant(s). However, the genotype–phenotype correlation of many variants remains unexplored. To date, several variants have been found to be clearly associated with CD-related thrombotic events, such as the p.Arg16Cys variant (Nanning), p.Ser532Cys (Caracas V), and p.Arg554Cys (Dusart) in Fib α-chain; p.Ala68Thr (Naples) in β-chain; and p.Asp364Val (Melun) in γ-chain (1, 10). Compared with heterozygous variants, homozygous and compound heterozygous variants in the Fib gene are more likely to cause bleeding/thrombotic symptoms (1, 2). However, it is important to note that the clinical presentation of CD can vary among individuals carrying the same variant, even within the same family.

In this study, we identified a heterozygous *FGG* c.952G>A (p.Gly318Ser; rs267606810) variant in the patient and his asymptomatic older sister. This variant occurs in the C-terminal of Fib γ-chain, which plays an important role in the structure and function of Fib. Fib proteins undergo a complex process called polymerization to form fibrin (11, 12). The C-terminal region of Fib γ-chain participates in polymerization by facilitating the proper alignment, association, and stabilization of Fib molecules (11, 12). In addition, the C-terminal region of the γ-chain contains binding sites for various proteins involved in coagulation and fibrinolysis processes, including factor XIIIa, thrombin, plasminogen, and tissue-type plasminogen activator (tPA) (11, 12). Therefore, it stands to reason that the p.Gly318Ser variant we have reported impairs the stability and functionality of the fibrin clot and the subsequent activation of fibrinolysis. *FGG* c.952G>A has been identified as a mutation associated with thrombotic diseases; however, a clear clinical phenotype remains to be defined (7). The ClinVar database lists p.Gly318Ser as a variant of uncertain significance. Here, we provide evidence that recurrent cerebral infarcts are clinical manifestations of this variant. However, further research is needed to evaluate the pathogenicity of *FGG* c.952G>A and investigate the underlying mechanism.

The Clauss method is commonly used to quantify the Fib concentration in plasma based on calculation of the conversion rate of Fib to fibrin (3). Due to abnormal Fib structure or function, the Clauss method often detects reduced plasma Fib levels in CD patients (6). However, in the same patients, plasma Fib may be determined to be normal or even increased when tested using the PT-derived method, which calculates Fib concentrations indirectly by measuring plasma turbidity during the PT clotting process (6). CD patients may be misdiagnosed as having hypofibrinogenemia when tested using the Clauss method or overlooked when diagnosed by the PT-derived method. Many studies have emphasized the combined use of these two laboratory tests to determine plasma Fib levels in CD patients (6, 13). It has been reported that the PT-derived/Clauss ratio of Fib (>1.43) has excellent sensitivity and specificity for the diagnosis of CD (6). In this study, we carefully measured the patient’s plasma Fib levels using both the Clauss method and the PT-derived method, and the PT-derived/Clauss ratio was 6.71, which is consistent with the characteristics of CD. However, it should be noted that both the Clauss method and the PT-derived method depend on the function of Fib. Immunologic detection of Fib antigens is still required to distinguish dysfibrinogenemia from hypofibrinogenemia. In our study, we found that the levels of Fib γ-chain in the plasma of our patient and his sister, who both carried the p.Gly318Ser variant, were higher than those of healthy controls.

Therapy for CD patients who undergo thrombotic events can be determined according to the specific clinical scenario. In general, anticoagulation therapy and/or antiplatelet therapy may be used initially to treat acute thrombotic events (1). These therapies may help to prevent further thromboembolism and allow the anticoagulants to dissolve the existing thrombus, even if this increases the risk of bleeding. In fact, anticoagulant therapy with rivaroxaban, a specific factor Xa inhibitor, showed an obvious side effect in the form of bleeding in our patient. Fortunately, dual antiplatelet therapy with aspirin and clopidogrel showed satisfactory long-term efficacy in the treatment of cerebral thrombosis, but aspirin alone did not seem to be able to control the recurrence of cerebral infarction. However, further extensive studies are needed to understand the efficacy and safety of antiplatelet agents in people with Fib gene variants.

In summary, we report a CD patient characterized by recurrent ischemic stroke, an uncommon clinical entity in CD. Family screening and genetic testing identified a heterozygous *FGG* c.952G>A (rs267606810) variant in this patient and his asymptomatic older sister with coagulation disorder. Our report expands the clinical phenotype spectrum of the *FGG* c.952G>A variant and underscores the importance of considering CD as a potential cause of unexplained ischemic stroke, especially for those with a family history of coagulation disorder.

## Data availability statement

The datasets presented in this article are not readily available because of ethical and privacy restrictions. Requests to access the datasets should be directed to the corresponding authors.

## Ethics statement

The studies involving humans were approved by the Ethics Committee of Taizhou Hospital, Zhejiang Province. The studies were conducted in accordance with the local legislation and institutional requirements. Written informed consent for participation in this study was provided by the participants’ legal guardians/next of kin. Written informed consent was obtained from the individual(s) for the publication of any potentially identifiable images or data included in this article.

## Author contributions

AY: Writing – original draft. YZ: Data curation, Writing – original draft. CW: Methodology, Writing – original draft. TW: Software, Writing – original draft. XZ: Formal analysis, Writing – review & editing. SW: Formal analysis, Writing – review & editing. SK: Data curation, Writing – review & editing. YB: Data curation, Writing – review & editing. YL: Writing – original draft. FW: Writing – original draft.

## Footnotes

^1^ https://www.ncbi.nlm.nih.gov/clinvar/RCV000851954

## References

[1] Casini A Neerman-Arbez M Ariens RA de Moerloose P. Dysfibrinogenemia: from molecular anomalies to clinical manifestations and management. J Thromb Haemost. (2015) 13:909–19. doi: 10.1111/jth.12916 25816717

[2] Tiscia GL Margaglione M. Human fibrinogen: molecular and genetic aspects of congenital disorders. Int J Mol Sci. (2018) 19:1597. doi: 10.3390/ijms19061597 29844251 PMC6032319

[3] Lebreton A Casini A. Diagnosis of congenital fibrinogen disorders. Ann Biol Clin. (2016) 74:405–12. doi: 10.1684/abc.2016.1167 27492693

[4] Chen X Yan J Xiang L Lin F. Misdiagnosis of a patient with congenital dysfibrinogenemia: a case report and literature review. J Clin Lab Anal. (2022) 36:e24624. doi: 10.1002/jcla.24624 35949040 PMC9459280

[5] Jia Y Zhang XW Wu YS Wang QY Yang SL. Congenital dysfibrinogenemia misdiagnosed and inappropriately treated as acute fatty liver in pregnancy: a case report and review of literature. World J Clin Cases. (2022) 10:12996–3005. doi: 10.12998/wjcc.v10.i35.12996 36569010 PMC9782930

[6] Xiang L Luo M Yan J Liao L Zhou W Deng X. Combined use of Clauss and prothrombin time-derived methods for determining fibrinogen concentrations: screening for congenital dysfibrinogenemia. J Clin Lab Anal. (2018) 32:e22322. doi: 10.1002/jcla.22322 28922493 PMC6816876

[7] Downes K Megy K Duarte D Vries M Gebhart J Hofer S. Diagnostic high-throughput sequencing of 2396 patients with bleeding, thrombotic, and platelet disorders. Blood. (2019) 134:2082–91. doi: 10.1182/blood.2018891192 31064749 PMC6993014

[8] Zhou J Ding Q Chen Y Ouyang Q Jiang L Dai J. Clinical features and molecular basis of 102 Chinese patients with congenital dysfibrinogenemia. Blood Cells Mol Dis. (2015) 55:308–15. doi: 10.1016/j.bcmd.2015.06.002 26460252

[9] Casini A Blondon M Lebreton A Koegel J Tintillier V de Maistre E. Natural history of patients with congenital dysfibrinogenemia. Blood. (2015) 125:553–61. doi: 10.1182/blood-2014-06-582866 25320241 PMC4296015

[10] Bor MV Feddersen S Pedersen IS Sidelmann JJ Kristensen SR. Dysfibrinogenemia-potential impact of genotype on thrombosis or bleeding. Semin Thromb Hemost. (2022) 48:161–73. doi: 10.1055/s-0041-1730358 34261148

[11] Pieters M Wolberg AS. Fibrinogen and fibrin: an illustrated review. Res Pract Thromb Haemost. (2019) 3:161–72. doi: 10.1002/rth2.12191 31011700 PMC6462751

[12] Weisel JW Litvinov RI. Fibrin formation, structure and properties. Subcell Biochem. (2017) 82:405–56. doi: 10.1007/978-3-319-49674-0_13 28101869 PMC5536120

[13] Miesbach W Schenk J Alesci S Lindhoff-Last E. Comparison of the fibrinogen Clauss assay and the fibrinogen PT derived method in patients with dysfibrinogenemia. Thromb Res. (2010) 126:e428–33. doi: 10.1016/j.thromres.2010.09.004 20947138

